# Identification of Endoplasmic Reticulum Stress-Related Subtypes, Infiltration Analysis of Tumor Microenvironment, and Construction of a Prognostic Model in Colorectal Cancer

**DOI:** 10.3390/cancers14143326

**Published:** 2022-07-08

**Authors:** Baike Liu, Xiaonan Yin, Guangfu Jiang, Yang Li, Zhiyuan Jiang, Liming Qiang, Na Chen, Yating Fan, Chaoyong Shen, Lei Dai, Yuan Yin, Bo Zhang

**Affiliations:** 1 Department of Gastrointestinal Surgery, West China Hospital, Sichuan University, Chengdu 610041, China; baike.liu@outlook.com (B.L.); yxnyinxiaonan@163.com (X.Y.); dr.jiangzhiyuan@foxmail.com (Z.J.); scyshenchaoyong@163.com (C.S.); 2 State Key Laboratory of Biotherapy and Cancer Center, West China Hospital, Sichuan University and Collaborative Innovation Center for Biotherapy, Chengdu 610041, China; cncncn0528@outlook.com (N.C.); saufan@163.com (Y.F.); dailei2016@scu.edu.cn (L.D.); 3 Department of Gastrointestinal Surgery, Guang’an People’s Hospital, Guang’an 638500, China; huaxi_jgf@outlook.com (G.J.); cnytlxx@hotmail.com (Y.L.); 4 Department of Gastroenterology Ward, Guang’an People’s Hospital, Guang’an 638500, China; huaxi_qlm@outlook.com; 5 School of Pharmacy, Chengdu Medical College, Chengdu 610500, China

**Keywords:** ER stress, colorectal cancer, tumor microenvironment, spatial transcriptomics, prognostic model

## Abstract

**Simple Summary:**

Understanding how endoplasmic reticulum stress influences colorectal cancer progression and the composition of the tumor microenvironment is important for developing novel strategies in the treatment of colorectal cancer. In this study, we identified two endoplasmic reticulum stress-related subtypes of colorectal cancer with distinct prognosis and infiltration patterns in the tumor microenvironment. Besides, we constructed a prognostic model for predicting patients’ survival, which involved an endoplasmic reticulum stress-related 14-gene signature. Furthermore, by utilizing spatial transcriptomics data from two untreated colorectal cancer patients, we explored endoplasmic reticulum stress-related gene signatures at a subcellular level and found that colorectal cancer cells and regulatory T cells showed an evidently increased expression of endoplasmic reticulum stress-related gene signature, and cancer-associated fibroblasts might be the leading characteristic that distinguishes the endoplasmic reticulum stress-related subtypes of colorectal cancer. We suggest that targeting endoplasmic reticulum stress in colorectal cancer might reshape the exhausted tumor microenvironment and mitigate tumor progression.

**Abstract:**

Recently, endoplasmic reticulum (ER) stress has been shown to influence tumor progression and immune cell function in the tumor microenvironment (TME). However, the underlying role of ER stress-related gene patterns in colorectal cancer (CRC) development remains unclear. We analyzed the ER stress-related gene patterns in 884 patients with CRC from the Gene Expression Omnibus database and evaluated the cell-infiltrating patterns in the TME. Two ER stress-related patterns were identified in patients with CRC that had distinct cell-infiltrating patterns in the TME and clinical characteristics. A risk score and nomogram based on 14 screened prognosis-correlated genes was built and validated to predict patient survival. Patients with a higher risk score were shown to have an unfavorable prognosis, and the risk score was associated with cell infiltration and drug sensitivity. Furthermore, spatial transcriptomics data were utilized to explore ER stress-related gene patterns in CRC tissues, and it was shown that ER stress phenotype involves in the formation of the immunosuppressive TME. This study demonstrated that ER stress-related gene patterns play a role in influencing the TME and predicting prognosis. These analyses of ER stress in the TME of CRC might deepen our understanding of CRC progression and immune escape and provide novel insights into therapeutic strategies.

## 1. Introduction

During the past decades, immunotherapies, such as immune checkpoint inhibitors (ICIs) and chimeric antigen receptor T cells (CAR-T), have obtained robust clinical responses and revolutionized the treatment of cancer patients [1]. Unfortunately, their efficacy varies, and only a limited number of patients benefit from immunotherapy, which is far from meeting the clinical demand [2,3,4]. It is widely accepted that different types of immune cells infiltrating the tumor microenvironment (TME) can influence tumor development and affect clinical outcomes [1,5,6]. Both innate immune cells (such as natural killer (NK) and dendritic cells (DC)) and adaptive immune cells (such as T and B cells) participate in tumor immunosurveillance. However, cancer cells can elicit multiple mechanisms to escape immunosurveillance or dampen antigen presentation, which necessitates immunotherapy that aims to boost the host immune system to eliminate tumor cells [1]. Moreover, the mechanisms underlying immunotherapy and tumor immune escape are not fully understood [7]. Understanding the TME and the mechanisms of immunotherapy may lead to novel therapeutic approaches for cancer.

The endoplasmic reticulum (ER) is one of the most vital organelles in eukaryotes and functions as a site of protein synthesis, folding, and modification, a calcium reservoir, and a compartment of lipid biosynthesis [8]. ER homeostasis is essential for normal cellular processes. ER stress can be induced under conditions such as high demand of secretion or protein synthesis, and is involved in many pathological diseases [9], including the aging process [10], neurodegenerative disease [11], and type 2 diabetes [12]. The unfolded protein response (UPR) pathway is the main mechanism for mitigating the induced ER stress in cells. To date, three parallel pathways have been discovered, and the three corresponding ER stress sensors located on the ER membrane are *inositol-requiring enzyme 1α* (*IRE1α*; also named *ERN1*), *PRKR-like endoplasmic reticulum kinase* (*PERK*; also named *EIF2AK3*), and *activating transcription factor 6* (*ATF6*) [13]. Recently, increasing evidence has shown that ER stress is involved in the regulation of cancer development and the function of immune cells in the TME [14,15].

Growing interest has been generated toward deciphering the role of ER stress-related pathways in the functional regulation of immune cells in the TME, albeit with conflicting results [13,16]. The ER stress sensor *IRE1α* and its substrate *XBP1* in NK cells are elevated to boost anti-tumor effects through their downstream target *c-Myc* [17]. The *IRE1α-XBP1* axis was also shown to be crucial in the tumor antigen cross-presentation of CD8α^+^ conventional DCs, and deficiency of *XBP1* in CD8α^+^ DCs resulted in a defective phenotype and antigen-presenting capacity [18]. In contrast, constitutive activation of *XBP1* in DCs results in abnormal accumulation of lipids in tumor-associated DCs and subsequent dysfunction of antitumor T cells in ovarian cancer [19]. Additionally, persistent activation of ER stress pathways in myeloid-derived suppressor cells (MDSC) has been shown to form an immunosuppressive environment and lead to tumor progression [13,20]. The studies mentioned above only focused on a restricted number of ER stress regulators, while the regulation of tumor development and the TME contains numerous factors that are highly coordinated. Thus, the ER stress patterns in cancer and the TME remain elusive and require further elucidation.

In this study, we integrated the transcriptomic data and clinical information of 1476 patients with colorectal cancer (CRC) to identify the relationship between ER stress-related regulators and immune cell infiltration characteristics, as well as clinical outcomes in CRC patients. We identified two ER stress-related sub-clusters (ERcluster A and ERcluster B), and based on the differentially expressed genes (DEGs) between the ERcluster A and ERcluster B, another two gene clusters (GeneCluster A and GeneCluster B) were identified. Next, we built a risk score system and nomogram based on the ER stress-related gene signatures to predict patient survival. Furthermore, we included spatial transcriptomic (ST) data to visualize ER stress-related gene patterns in cell subtypes.

## 2. Materials and Methods

### 2.1. Data and Resources

Microarray gene expression data and clinical information including age, gender, tumor location, stage, *KRAS* mutation, and *BRAF* mutation of CRC tumor samples (GSE17536, GSE38832, and GSE39582, *n* = 884), were downloaded from the Gene Expression Omnibus (GEO) (https://www.ncbi.nlm.nih.gov/geo/, accessed on 1 November 2021). The above three GEO datasets were all based on the GPL570 platform; other platforms were not accepted because certain uncontrollable biases might be included. The Cancer Genome Atlas (TCGA) Colon Adenocarcinoma (COAD)/Rectum adenocarcinoma (READ) expression data in fragments per kilobase of transcript per million mapped fragments (FPKM) and their corresponding clinical data were downloaded from the Genomic Data Commons (GDC) portal (https://portal.gdc.cancer.gov/, accessed on 1 November 2021). The gene expression FPKM value of TCGA COAD/READ was transformed into the TPM format. Three independent cohorts of GEO datasets were combined, and batch effects were removed using the R package “Combat”. Patients with missing survival information were excluded from the study. Thus, 884 patients from the GEO database and 592 patients from the TCGA database were included in this study.

### 2.2. Unsupervised Clustering Analysis of ER Stress-Related Genes

The ER stress-related gene set (GOBP_RESPONSE_TO_ENDOPLASMIC_RETICULUM_STRESS) was downloaded from the Molecular Signatures Database v7.5.1 (MSigDB, http://www.gsea-msigdb.org/, accessed on 1 November 2021). A total of 295 genes were included in the GOBP_RESPONSE_TO_ENDOPLASMIC_RETICULUM_STRESS gene set, 268 of which were found in GEO and TCGA expression data (Supplementary Table S3). Unsupervised consensus clustering was realized by the R package “ConsensusClusterPlus” to find the molecular subtypes of CRC; repetitions were set at 1000 to make sure the stability of clustering. The optimal number of clusters was determined using the cumulative distribution function (CDF) curve, which showed a gently increasing trend. Furthermore, to examine the differences in the biological processes between the ER stress-related sub-clusters, gene set variation analysis (GSVA) was utilized, and the gene set c2. cp.kegg.v7.2, downloaded from MSigDB, and analysis was performed using the R package “GSVA” [21].

### 2.3. Estimation of Lymphocyte Infiltration in the Bulk Sequencing Data

Several algorithms, including single-sample gene set enrichment analysis (ssGSEA) [21], microenvironment cell population (MCP)-counter [22], and CIBERSORT [23], have been used to reflect and cross-validate the infiltration of different immune cell subtypes. The 28 immune cell signatures applied in ssGSEA were derived from Charoentong et al. [24], and the ESTIMATE algorithm was used to assess the immune and stromal scores of each tumor sample [25]. The patients were then grouped based on the clusters to identify differences in lymphocyte infiltration between the clusters.

### 2.4. Biological Functions and Pathways Enrichment Analysis

Differentially expressed genes (DEGs) between the ER stress-related subtypes were identified with the “limma” package in R, fold change value was set at 0.8, and *p*-value < 0.05 was accepted as statistically significant [26] (Supplementary Table S4). Another R package, “clusterProfiler, ” was used to evaluate the biological functions and pathways of these ER stress-related DEGs [27]. Gene Ontology (GO) and Kyoto Encyclopedia of Genes and Genomes (KEGG) pathway analyses were performed with a *p*-value and an adjusted *p*-value of <0.05.

### 2.5. Construction of ER Stress-Related Risk Score

The ER stress-related risk score was calculated to quantify individual tumor types and identify patient prognosis. Univariate Cox analysis was performed on 268 genes from the GOBP_RESPONSE_TO_ENDOPLASMIC_RETICULUM_STRESS gene set to determine those correlated with patient survival. Genes (*n* = 232, Supplementary Table S2) correlated with prognosis in the former step were included in the Lasso and multivariate Cox regression analyses. Patients with CRC were randomly divided into training set and testing set at a ratio of 1:1; for these processes, the “glmnet” package in R software was utilized. The model was determined by the smallest lambda value according to 10-fold cross-validation, and the ER stress-related risk score was formulated as follows: Risk score = Σ (Expi ×Coef) where Expi and Coef represent the gene expression value and risk coefficient, respectively (Table 1). The median risk score was set as the cutoff value to divide the patients into high-risk (risk score > median) and low-risk (risk score < median) groups. In addition, using the package “ggplot2” in R software, principal component analysis (PCA) was performed based on ER stress-related genes. The correlation between the risk score and lymphocyte infiltration results of CIBERSORT was conducted using Pearson’s correlation analysis in R. To evaluate the sensitivity of common chemotherapeutic drugs between the two risk groups, another R package “pRRophetic” was used to calculate the half-maximal inhibitory concentration (IC50) values of these drugs and visualized in boxplot. Analysis of protein-protein interaction (PPI) networks among the above 232 prognostic genes was conducted using STRING database (11.0) [28]. MCODE algorithm [29] was utilized to find complex modules within large protein interaction networks and the results were further visualized in Cytoscape [30].

### 2.6. Mutation, Copy number Variation, and DNA Methylation Analysis

R package “GenVisR” was used to visualize the gene mutational landscape. The copy number variation (CNV) landscape was plotted with the “RCircos” R package. For DNA methylation analysis, a web-based user-friendly tool named Shiny Methylation Analysis Resource Tool (http://www.bioinfo-zs.com/smartapp/, accessed on 1 November 2021) was used to comprehensively analyze the DNA methylation levels of ER stress-related genes [31] in TCGA projects.

### 2.7. Real-Time Quantitative PCR Analysis

Frozen CRC tumor samples and their paired adjacent normal tissues from six patients were acquired from Sichuan University West China Hospital. Total RNA was extracted using the TRIzol reagent (Invitrogen, Carlsbad, CA, USA). The PrimeScript^TM^ II 1st Strand Synthesis Kit (Takara, Kusatsu, Japan) was used for reverse transcription to convert RNA into cDNA, and the procedures were performed according to the manufacturer’s instructions. Real-time quantitative PCR (RT-qPCR) was performed on a QuantStudio3 PCR system (Thermo Fisher Scientific, Waltham, MA, USA), and relative mRNA abundance was calculated using the 2^−^^∆∆Ct^ method. The forward and reverse primer sequences used for RT-qPCR are shown in Supplementary Table S5. This study was approved by the Ethics Committee of Sichuan University West China Hospital, Chengdu, China (approval number: 2018 (280)). Written informed consent was obtained from all patients before being enrolled in the study and the study was conducted in accordance with the principles of the Declaration of Helsinki.

### 2.8. Construction of a Nomogram in Predicting Survival

The “rms” package in R was applied and the risk score combined with other clinical factors such as stage and age which are associated patients’ prognosis were taken into account and incorporated into the establishment of a nomogram [32]. A calibration plot illustrating the observed and predicted survival rates was used to assess the performance of the established nomogram. Each variable was matched to a certain score according to the nomogram. The total score for each patient represents the predicted survival probability. Time-dependent receiver operating characteristic (ROC) curves for 1-, 3-, 5-, and 10-year survival were generated to verify the predictive ability of the nomogram system.

### 2.9. Spatial Transcriptomics Data Analysis

Two slides of untreated CRC tissue with spatial transcriptomics (ST) data were collected from a study by Wu et al. [33] (http://www.cancerdiversity.asia/scCRLM, accessed on 1 November 2021). Detailed procedures performed on tumor slides have been described by Wu et al. In the cell subtype annotation, each spot on the ST data contains several cells, and some existing overlapping cell markers might not illustrate the cell subtypes sufficiently. Thus, we applied several cell gene markers from Charoentong et al. [24] and Racle et al. [34] to score the cell subtypes in the ssGSEA analysis.

### 2.10. Statistical Analysis

Statistical analyses were conducted using R software (version 4.0.5, Auckland, New Zealand). The Kruskal–Wallis test was used to compare lymphocyte infiltration scores and gene expression levels. Chi-square tests were used to evaluate the relationship between clinical characteristics (including age, sex, stage, grade, MSI status, KRAS mutation, and BRAF mutation) and ER stress-related sub-clusters. Kaplan–Meier and log-rank test were utilized to investigate overall survival (OS) between different sub-clusters and optimal cutoff value of risk score in TCGA COAD/READ was determined by the “survminer” and “survival” packages in R software. In addition, we applied univariate and multivariate Cox regression models to calculate the hazard ratio (HR) of ER stress-related genes in patients with CRC. For all statistical analyses, a two-tailed *p*-value < 0.05 was considered statistically significant.

## 3. Results

### 3.1. Identification of ER Stress Related Sub-Clusters in CRC

**Figure 1:**
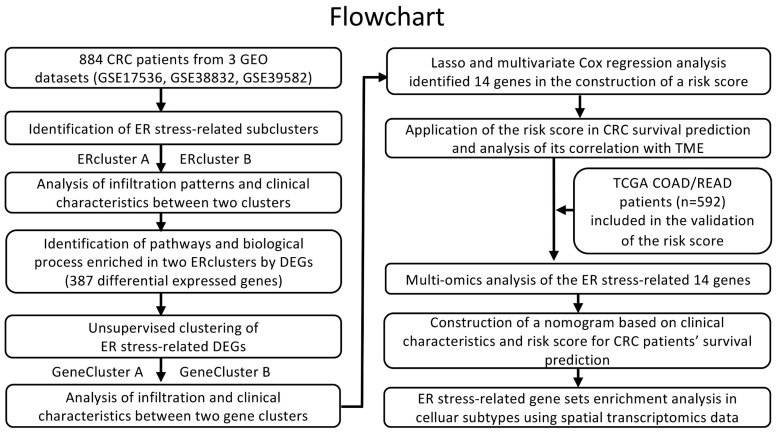
Overview of the analysis workflow.

**Figure 2:**
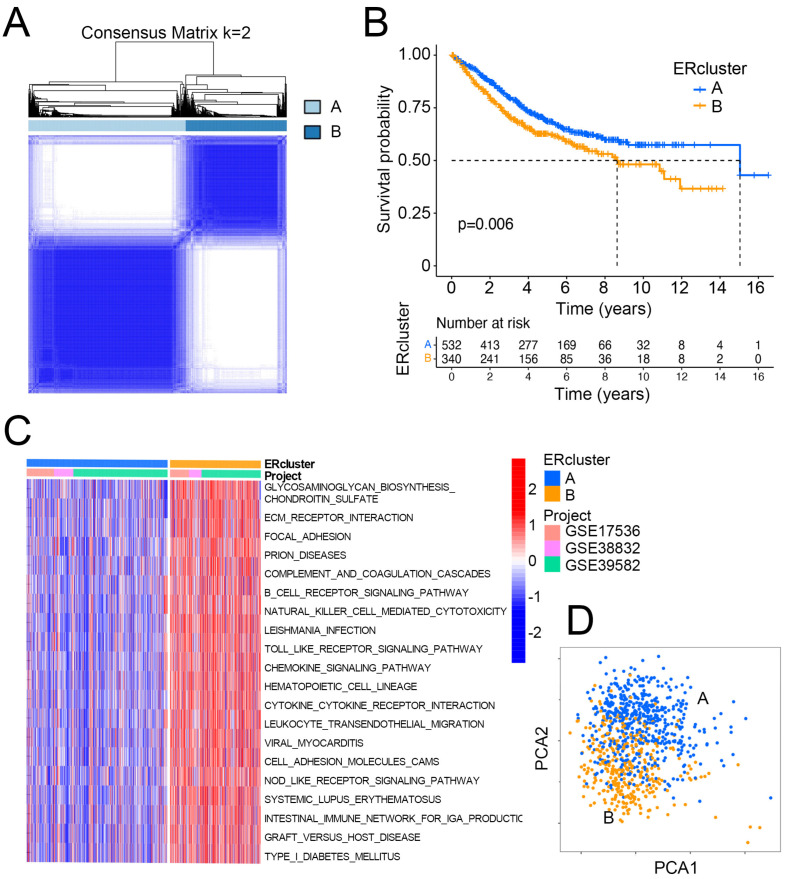
ER stress-related sub-clusters of CRC. (**A**) Consensus clustering defines two (k = 2) clusters named as ERcluster A (*n* = 532) and ERcluster B (*n* = 340) in patients with CRC (GSE17536, GSE38832, GSE39582) based on ER stress-related gene sets. (**B**) Kaplan–Meier survival analysis of the ERcluster A and B (log rank test *p* value = 0.006), x axis indicates the number of years after initial diagnosis of CRC. (**C**) GSVA analysis of pathways in the two sub-clusters, red and blue represent the upregulated and downregulated pathways in each sample, respectively. (**D**) PCA analysis illustrates a distinct difference of transcription profile between the two sub-clusters.

The entire analytical pipeline used in this study is shown in Figure 1. To gain deeper insight into the expression pattern of ER stress-related genes in CRC oncogenesis, 884 patients from three independent datasets from GEO (GSE17536, GSE38832, and GSE39582) were included. Using a consensus clustering algorithm based on the aforementioned 268 ER stress-related genes in two MSigDB gene sets (Supplementary Figure S1), k = 2 was found to be an optimal parameter for dividing the entire GEO CRC cohort into two sub-clusters, namely ERcluster A and ERcluster B (*n* = 538 and *n* = 346, respectively; Figure 2A). We further analyzed the clinical characteristics of the two sub-clusters (Table 2). It appeared that ERcluster B was preferably located at the proximal site, had a higher BRAF mutational rate, and a higher mismatch repair deficiency (dMMR) rate. No significant disparities were detected in terms of age, sex, stage, grade, *KRAS* mutational status, or adjuvant therapy. Survival analysis showed that ERcluster B had a poorer overall survival rate than ERcluster A (Figure 2B, log-rank *p* = 0.006). Furthermore, GSVA revealed that several immune-related pathways were enriched in ERcluster B, including B cell receptor signaling pathways, NK cell mediated cytotoxicity, and cytokine signaling pathways (Figure 2C). Subsequently, by applying the ER stress-related genes in the PCA analysis, the above two ER stress-related sub-cluster patterns could be well distinguished (Figure 2D). In general, the above results demonstrated that two different expression patterns of ER stress-related genes participated in CRC tumorigenesis and were correlated with patient prognosis.

### 3.2. ERcluster A and B Had Distinct Cell-Infiltrating Patterns in the TME

**Figure 3:**
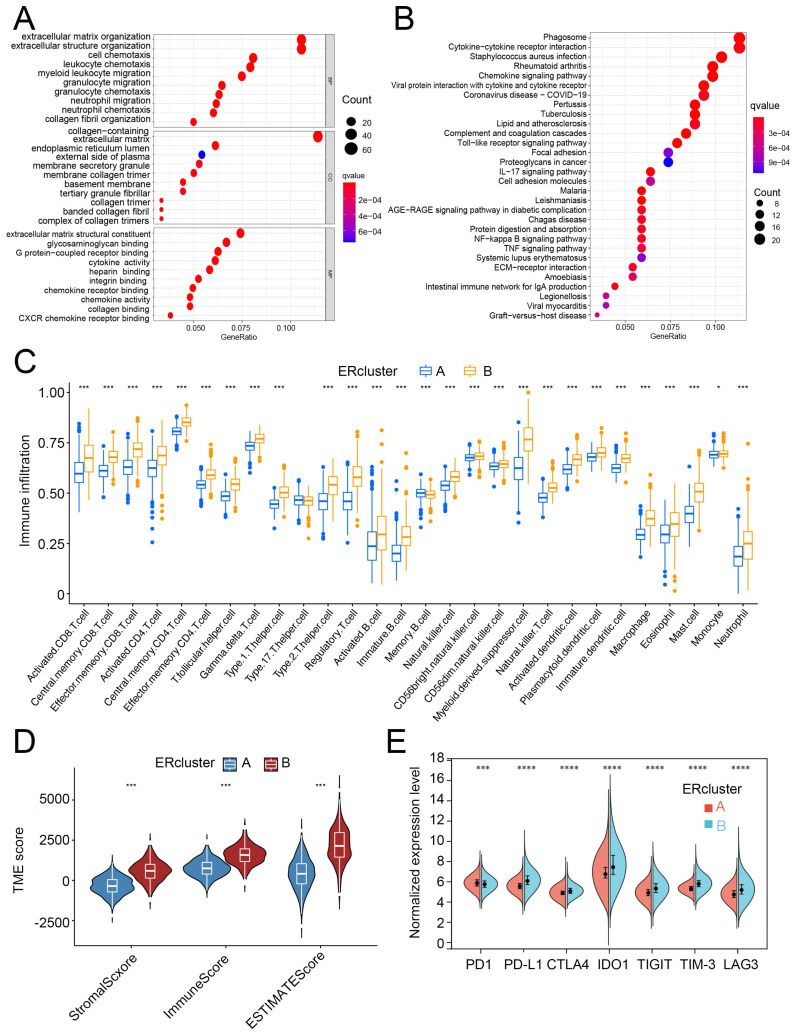
Functional analysis and the TME cell infiltration patterns between ERcluster A and B. (**A**,**B**) GO and KEGG pathways analysis of the identified 387 DEGs (Fold change value > 0.8 and *p*-value < 0.05). (**C**) The estimated infiltration abundance of 28 immune cells in ERcluster A and B. The upper and lower ends of the box represent the interquartile range, central bar represents the median value, and the dots stand for outliers (* *p* < 0.05, *** *p* < 0.001). (**D**) ESTIMATE scoring results of ERcluster A and B (* *p* < 0.05, *** *p* < 0.001). (**E**) Normalized expression level of common immune checkpoint genes (*PD1, PD-L1, CTLA4, IDO1, TIGIT, TIM-3,* and *LAG3*) between ERcluster A and B (* *p* < 0.05, *** *p* < 0.001, **** *p* < 0.0001).

To further detect the dominant pathways that separate ERcluster A and ERcluster B, 387 DEGs screened out by R package “limma” were used in GO and KEGG functional enrichment analysis (Figure 3A,B). These 387 ER stress-related DEGs were significantly enriched in immunological processes (Figure 3A). KEGG analysis revealed that several immunological and cancer-associated pathways were enriched (Figure 3B). Since the above results correlated ER stress-related genes with immunological events, we applied ssGSEA to analyze differential infiltration patterns in ERcluster A and B. As a result, cluster B was found significantly associated with immune cell infiltration, including CD8+ T cells, CD4+ T cells, Treg (regulatory T cells), DCs, and macrophages (Figure 3C). In the MCP-counter algorithm, which was designated in the absolute quantification of eight immune and two stromal cells, a similar infiltration pattern was found in ERcluster B (Supplementary Figure S2A). Higher infiltration of T cells, cytotoxic lymphocytes, monocytic lineage, and myeloid DCs, but not CD8+ T cells, was identified in ERcluster B. Additionally, stromal and endothelial cells and fibroblasts were more abundant in ERcluster B than in ERcluster A (Supplementary Figure S2A). Likewise, according to the ESTIMATE algorithm, ERcluster B exhibited both higher stromal and immune scores, which is consistent with previous results (Figure 3D). Furthermore, considering the crucial role of immune checkpoint genes in the negative regulation of tumor immunity (*PD1, PD-L1, CTLA4, IDO1, TIGIT, TIM-3,* and *LAG3*), we analyzed the expression of these genes and found that except for PD1, all other immune checkpoint genes were upregulated in ER cluster B (Figure 3E). In summary, ERcluster A and B showed distinct cell-infiltrating patterns in the TME. ERcluster B seemed to have more immune cells, stromal cell infiltration, and higher expression of common immune checkpoint genes.

### 3.3. ER Stress-Related Gene Clusters Had Distinct Clinical Characteristics and Cell-Infiltration Patterns in the TME

**Figure 4:**
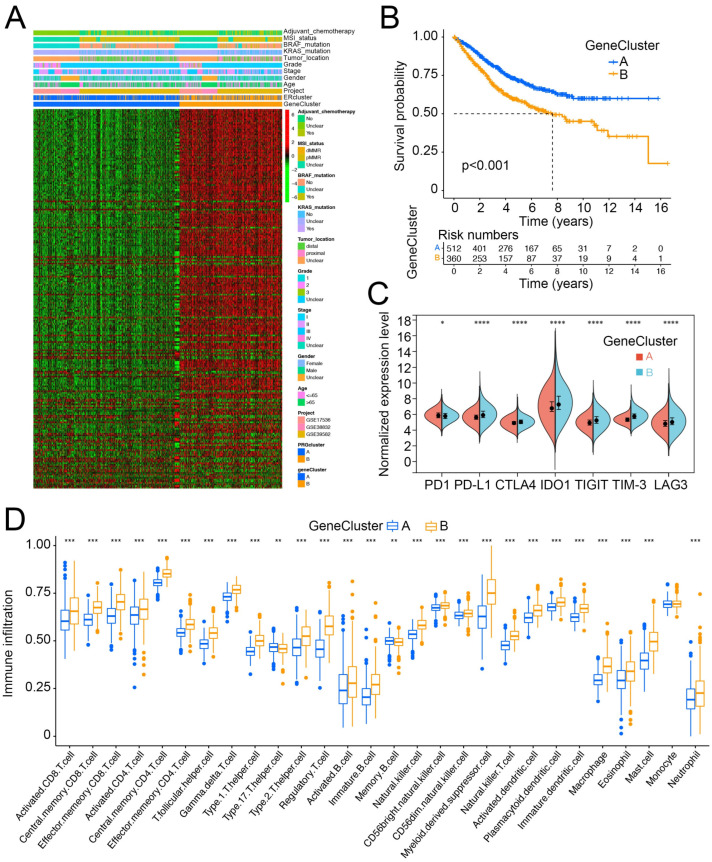
Expression profile of two sub-clusters based on the 387 DEGs (named as GeneCluster A and GeneCluster B) and their immune infiltration characterization. (**A**) Expression profile of 387 DEGs and clinical characteristics of the two gene clusters. (**B**) Kaplan–Meier survival analysis of the GeneCluster A and B (log rank test *p* value < 0.001), x axis indicates the number of years after initial diagnosis of CRC. (**C**) Expression level of common immune checkpoint genes (*PD1, PD-L1, CTLA4, IDO1, TIGIT, TIM-3,* and *LAG3*) between GeneCluster A and B (* *p* < 0.05, ** *p* < 0.01, *** *p* < 0.001, **** *p* < 0.0001). (**D**) The estimated infiltration abundance of 28 immune cells in GeneCluster A and B (* *p* < 0.05, ** *p* < 0.01, *** *p* < 0.001).

Based on the 387 ER stress-related DEGs, we conducted unsupervised clustering to further explore the heterogeneity of ER stress-related subtypes. The 884 patients with CRC were also divided into two gene clusters, GeneClusters A and B (Figure 4A, Supplementary Figure S3). The clinical features of GeneClusters A and B resemble the difference between ERcluster A and B. In addition to a higher proportion of BRAF mutations, and dMMR and proximal tumor ratios in GeneCluster B, GeneCluster B also had a higher percentage of patients with stage III/IV disease (Supplementary Table S1). Survival analysis revealed that GeneCluster B tended to have a poor OS rate (Figure 4B). Furthermore, analysis of immune checkpoint genes and immune cell infiltration in the two GeneClusters showed similar patterns. The expression of *PD-L1, CTLA4, IDO1, TIGIT, TIM-3,* and *LAG3* was elevated in GeneCluster B, and more immune cell subtypes were also enriched (Figure 4C, D, Supplementary Figure S2B). In summary, based on the 387 ER stress-related DEGs, we further identified two subtypes of CRC (named GeneCluster A and B), which could better illustrate the different characteristics of ER stress-related subtypes. These two subtypes exhibit distinct clinical characteristics and cell infiltration patterns in the TME.

### 3.4. Construction and Validation of Risk Score and Its Clinical Significance

**Figure 5:**
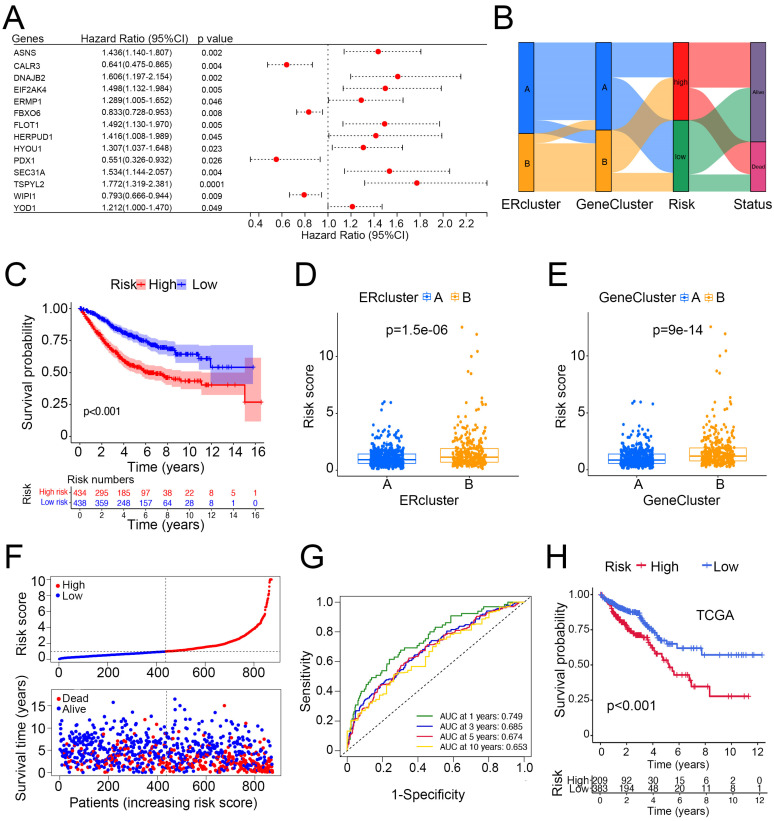
Construction of an ER stress-related risk score in predicting survival of patients with CRC. (**A**) Univariate Cox regression results of the 14 risk score-building genes in patients with CRC from GEO datasets. (**B**) Alluvial diagram of sub-cluster distributions with distinct risk score and clinical outcomes. (**C**) Survival analysis of the high- and low-risk group (log rank test *p* value < 0.001). (**D**,**E**) Difference of risk score in ERclusters and GeneClusters. (**F**) Ranked plots and distribution plots of risk score and clinical outcomes, respectively (median value of risk score was set as cutoff value). (**G**) ROC curves of the sensitivity and specificity of 1-, 3-, 5-, and 10-year survival prediction using risk score. (**H**) Validation of risk score in predicting TCGA COAD/READ survival (*n* = 592).

We sought to build a risk score based on ER stress-related genes to better depict the prognosis of CRC patients. First, 268 genes in the MSigDB gene sets were included in the univariate Cox analysis among patients with CRC from three independent GEO datasets (*n* = 884). A total of 232 genes within the gene list were found to have a prognostic value. Second, we randomly divided the patients into training (*n* = 442) and testing (*n* = 442) groups at a 1:1 ratio. Then, by applying the 232 genes in the LASSO and multivariate Cox regression analyses, 14-gene ER stress-related signatures (*ASNS*, *CALR3*, *DNAJB2, EIF2AK4*, *ERMP1*, *FBXO6*, *FLOT1*, *HERPUD1*, *HYOU1*, *PDX1*, *SEC31A*, *TSPYL2*, *WIPI1*, and *YOD1*) were screened to build the ER stress-related risk score, and the median value was set as the cut-off point (Figure 5A, Supplementary Figure S4A,B). Those with a risk score above the median value were included in the high-risk group and the other half, below the median value, was designated as the low-risk group. Figure 5B illustrates the distribution of the two ER stress-related clusters, gene clusters, risk groups, and endpoint events. According to the survival analysis, the high-risk group had a significantly poorer OS compared with the low-risk group (Figure 5C,F). Unsurprisingly, both ERcluster B and GeneCluster B had higher risk scores (Figure 5D,E). Additionally, the ER stress-related risk score showed a good predictive value in CRC for the time-dependent survival of patients with CRC. The area under the curve (AUC) values of the 1-, 3-, 5-, and 10-year survival rates of CRC patients were 0.749, 0.685, 0.674, and 0.653, respectively (Figure 5G). Moreover, to validate the prognostic value of the ER stress-related risk score, we utilized another cohort of CRC patients from the TCGA database (*n* = 592), and the result was consistent in that the high-risk group had a worse prognosis (Figure 5H).

In terms of the correlation of risk score and immune infiltration, we found that the risk score was positively correlated with the infiltration of macrophage M2, memory B cells, and macrophage M0. In contrast, the risk score was negatively correlated with CD8 T and activated CD4 memory T cells, and M1 macrophages (Supplementary Figure S5). In addition, we found that the risk score was associated with sensitivity to common chemotherapeutic drugs (Supplementary Figure S6). In summary, we constructed a risk score based on 14 ER stress-related genes and found that it can effectively distinguish poor prognosis among CRC patients. Additionally, the risk score was associated with immune cell infiltration and drug sensitivity.

### 3.5. Mutation, CNV, Transcription, and Methylation Level of the 14 Risk Score-Building Genes

**Figure 6:**
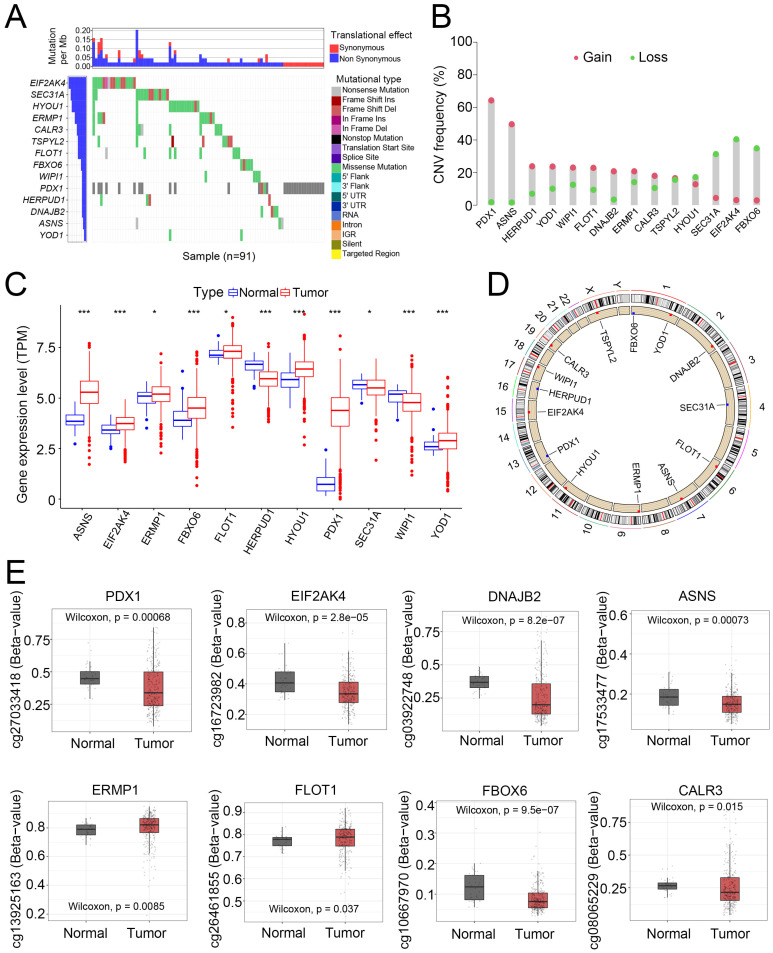
Mutational, transcriptional, and methylation alteration of the screened out 14 ER stress-related genes. (**A**) Mutational landscape of the 14 ER stress-related genes in 91 patients with CRC from TCGA database. (**B**) Frequencies of CNV gain and loss among the 14 genes. (**C**) DEGs among the 14 genes between CRC and normal adjacent tissue (* *p* < 0.05, *** *p* < 0.001). (**D**) Illustration of the locations of CNV gain and loss in the 14 ER stress-related genes. (**E**) Differentially methylated promoter sites among the 14 genes.

After sorting the 14 genes to build the risk score, we further analyzed these key genes at the multi-omics level. Among these genes, *EIF2AK4* had the highest mutation frequency, followed by *SEC31A*, *HYOU1*, and *ERMP1* (Figure 6A). Next, we evaluated CNV in these genes. *PDX1* and *ASNS* showed a widespread CNV increase, while *SEC31A*, *EIF2AK4*, and *FBOX6* showed CNV loss (Figure 6B). As for the transcriptomic level, *ASNS*, *EIF2AK4*, *ERMP1*, *FBOX6*, *FLOT1*, *HYOU1*, *PDX1*, and *YOD1* were significantly elevated in tumor samples, while *HERPUD1* and *WIPI1* were downregulated (Figure 6C). Using the six pairs of frozen CRC tumors and their normal adjacent samples, we validated that *PDX1* and *YOD1* expression was significantly increased in CRC tumors and that *HERPUD1* and *DNAJB2* were suppressed. Other genes, such as *ASNS*, *EIF2AK4*, *ERMP1*, and *HYOU1*, showed an increasing trend in tumor tissue, but this was not statistically significant (Supplementary Figure S7). Figure 6D illustrates the locations of CNV in the 14 genes on their corresponding chromosomes.

Furthermore, DNA methylation analysis revealed that specific loci in *PDX1*, *EIF2AK4*, *ASNS*, *DNAJB2*, and *FBOX6* were hypomethylated in tumor samples (Figure 6E). In general, the multi-omics data were consistent. The increased expression of *PDX1* and *ASNS* might be due to CNV gain and DNA hypomethylation, indicating that CNV and DNA methylation might regulate the expression of ER stress-related genes. However, *EIF2AK4* and *FBOX6* showed loss of CNV, but their expression was increased, whereas other genes with increased or decreased CNV or differentially methylated DNA loci showed no expression disparities between normal and tumor tissues. Thus, we speculated that CNV and DNA methylation might participate in the regulation of 14 ER stress-related genes. Other mechanisms, such as transcription factors and histone acetylation, could also be involved in the regulation of these genes. In summary, these results demonstrated evident multi-omics changes (including the mutation landscape, CNV, expression, and methylation level) in the 14 ER stress-related genes in CRC, indicating that these genes and the ER stress-related gene pattern might be involved in CRC carcinogenesis.

### 3.6. Development and Verification of a Nomogram in Predicting Survival of Patients with CRC

**Figure 7:**
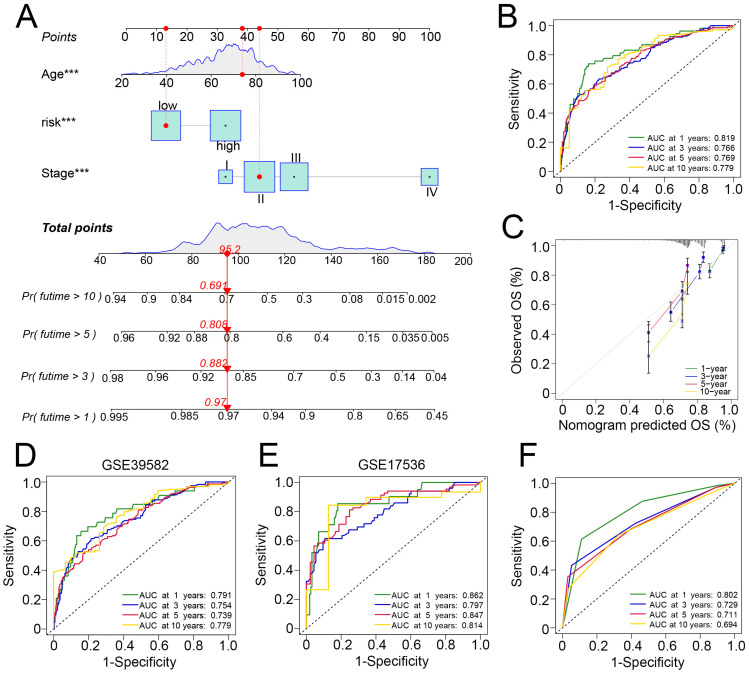
Development and validation of a nomogram. (**A**) Nomogram for the 1-, 3-, 5-, and 10-year survival prediction in CRC, *** indicate *p* < 0.001 in the univariate Cox proportional hazards regression analysis. (**B**) Time-dependent ROC curve of the nomogram for 1-, 3-, 5-, and 10-year survival prediction in the overall GEO patients with CRC. (**C**) Calibration plot for internal validation of the nomogram in the overall GEO patients with CRC. (**D**,**E**) ROC curve of the nomogram for 1-, 3-, 5-, and 10-year survival prediction in the subgroups, GSE17536 and GSE39582. (**F**) ROC curve of the TNM stage for 1-, 3-, 5-, and 10-year overall survival prediction.

Because the inconvenience and consideration of other clinical features such as age and tumor stage were correlated with tumor prognosis, we incorporated these clinicopathological features into the construction of a nomogram to predict the 1-, 3-, 5-, and 10-year survival of patients with CRC (Figure 7A). In all GEO patients with CRC, the AUC values of the nomogram were 0.819, 0.766, 0.769, and 0.779 for the 1-, 3-, 5-, and 10-year survival predictions, respectively. The calibration curve showed that the nomogram was capable of predicting prognosis (Figure 7B,C). We tested the nomogram on the GEO subgroups. In GSE17536, the 1-, 3-, 5-, and 10-year AUC values were 0.862, 0.797, 0.847, and 0.814, respectively. In GSE39582, the 1-, 3-, 5-, and 10-year AUC values were 0.791, 0.754, 0.739, and 0.779, respectively (Figure 7D,E). To compare the performance of the nomogram with that of simply using TNM stage for survival prediction, we found that the AUC values of the TMN stage were 0.802, 0.729, 0.711, and 0.694 for 1-, 3-, 5-, and 10-year survival, respectively, in patients with CRC (Figure 7F). These results suggest that the nomogram had a superior ability to predict the survival of patients with CRC and might be utilized in clinical practice.

### 3.7. Insights into ER Stress-Related Gene Signatures at the ST Level

**Figure 8:**
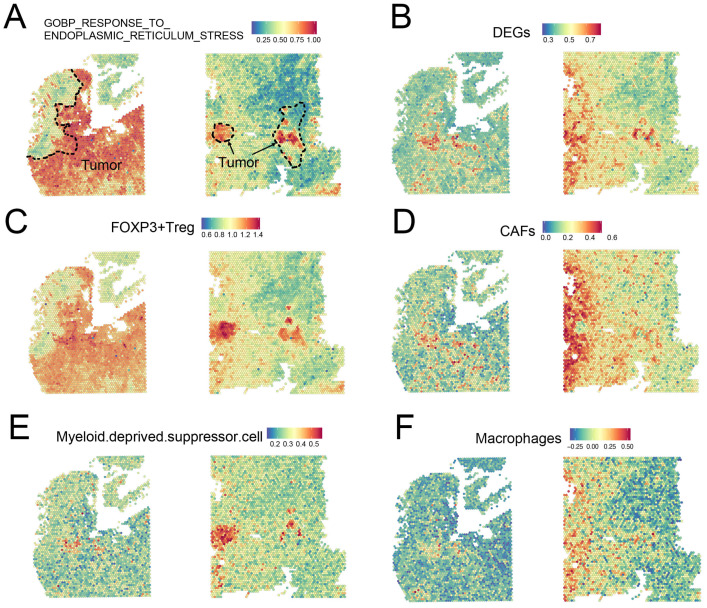
Mapping of gene signatures ER stress and immune cells on CRC ST data. (**A**) The enrichment score of GOBP_RESPONSE_TO_ENDOPLASMIC_RETICULUM_STRESS gene set on ST data; the dotted line represents the tumor region. (**B**) Enrichment score of DEGs between ERcluster A and B on ST data. (**C**–**F**) Enrichment score of FOXP3+ Treg, CAFs, MDSC, and Macrophages signatures on ST data.

Although we identified two ER stress-related heterogeneous subgroups of patients with CRC with discrepant prognoses, there was still a huge gap in understanding the extent to which these ER stress-related gene signatures were enriched and what cell subtypes were preferentially influenced by these gene signatures. Thus, we employed ST data to identify these gene signatures, not only in terms of their expression, but also at the spatial level. Two untreated CRC tissue sections and their corresponding transcriptomic data were gathered from the web portal developed by Wu et al. [33] (http://www.cancerdiversity.asia/scCRLM, accessed on 1 November 2021). Next, using the ssGSEA method as described by Wu et al. [33], two gene signatures, GOBP_RESPONSE_TO_ENDOPLASMIC_RETICULUM_STRESS (abbreviated as ER stress signature) and DEGs between ERcluster A and B (shortened as DEGs signature) were calculated and visualized in the ST data (Figure 8A,B). We found that the ER stress signature was highly enriched in the tumor region compared to the nearby normal region. This indicates that the tumor cells and their surrounding microenvironment were highly influenced by ER stress patterns or that the ER stress-related genes participated in tumor biological processes. In addition, the FOXP3+ Treg region was found to highly overlap with ER stress signatures (Figure 8C). More interestingly, using the DEGs between ERcluster A and B as a gene list in ssGSEA, we found that the DEGs signature was highly enriched in the region of cancer-associated fibroblasts (CAFs) (Figure 8D). MDSC and macrophage signatures also seemed to overlap with the DEGs and ER stress signatures (Figure 8E,F). As for CD4+ and CD8+ T cells, their correlation with the above two signatures was not evident (data not shown). Given the above, these results demonstrated that the ER stress gene pattern was associated with lymphocyte infiltration, especially FOXP3+ Tregs and MDSC, and the DEGs were significantly associated with CAFs and macrophages, which might provide insight as to why ERcluster B and GeneCluster B have a poorer survival (both ERcluster B and GeneCluster B have a higher enrichment score in ER stress and DEGs signatures, data not shown), since previous studies showed that CAF-secreted exosomes and macrophages might be associated with poorer prognosis, tumor progression, and epithelial-mesenchymal transition [35,36,37].

## 4. Discussion

Increasing evidence has demonstrated that ER stress-related genes play a crucial role in shaping the TME, influencing the function of immune cells in the TME and tumor development [14,15]. However, most studies have only focused on single immune cell types and a few ER stress regulators. The overall transformation of immune cells in the TME, mediated by multiple ER stress-related regulators, remains largely unexplored. In addition, analysis of ER stress-related gene patterns in CRC development is also lacking. Thus, in this study, we included multiple ER stress-related genes in the analysis of CRC, which might deepen our understanding of the role of ER stress in shaping the CRC TME and help develop novel therapeutic strategies to boost the efficacy of immunotherapy in CRC.

Considering CRC as a rather heterogenic cancer type [38], we applied the ER stress-related gene signature in the unsupervised clustering and identified two sub-clusters of CRC, which were named ERcluster A and ERcluster B. To further explore the distinct characteristics and biological functions between these two subtypes, we applied the DEGs of ERcluster A and B in the unsupervised clustering and found another two sub-clusters of CRC, named GeneCluster A and GeneCluster B. These subtypes exhibit distinct clinical characteristics, such as tumor locations and MSI status. According to the survival analysis, we found that ERcluster B and GeneCluster B showed inferior prognosis compared with ERcluster A and GeneCluster A, which suggests that ER stress-related gene patterns might participate in tumor progression, since ERcluster B and GeneCluster B had a higher enrichment score in the ER stress signature. Previous studies have demonstrated that ER stress-induced activation of the UPR is a double-edged sword [14,39]. Under moderate ER stress, cancer cells are adaptive to intrinsic or extrinsic stimuli, which favors tumor progression, metastasis, and chemoresistance. The *IRE1α-XBP1* axis is elevated in triple-negative breast cancer (TNBC) [40]. *XBP1* aggravates *hypoxia-inducible factor 1α* (*HIF1α*) and activates its downstream pathways, leading to tumor angiogenesis and increased energy supply to support tumor progression and recurrence [40,41]. Under extreme ER stress, the outcome of tumor cells tended to be cell death. The *PERK-ATF4* axis and its downstream target, transcription factor *C/EBP homologous protein* (*CHOP*, also named *DDIT3*), were shown to be pro-apoptotic and cause cell death [42,43]. According to our results, overall ER stress-related gene patterns seem to allow cancer cells to adapt and thrive and are harmful to patient prognosis. However, the effect of ER stress was not confined within cancer cells, it could also be “transmissible” from cancer cells to surrounding immune cells or directly activated in immune cells by hostile milieu [15,44,45,46].

We further explored the tumor-infiltrating cells in these different ER stress-related subtypes. The results revealed that the two corresponding sub-clusters in ERclusters and GeneClusters had distinct cell-infiltrating patterns in the TME. Both ERcluster B and GeneCluster B significantly enriched innate and adaptive immune cells. Meanwhile, stromal cells, such as CAFs, were increasingly infiltrating in ERcluster B and GeneCluster B. Of note, among the immune cells, MDSC, regulatory T cells, and macrophages were elevated in ERcluster B and GeneCluster B with a much higher magnitude, indicating a poor prognosis in cancer patients [47,48]. The underlying mechanism of the distinct immune-infiltration patterns between ERcluster A and ERcluster B (GeneCluster A and GeneCluster B) might be caused by intrinsic CRC heterogeneity regarding different ER stress patterns. However, the role of different tumor origins could not be ruled out, as previous studies have shown proximal and distal CRC with different embryological origins have distinct cell-infiltrating patterns [49,50]. We then analyzed the immune checkpoint genes such as *PD-L1*, *CTLA4*, *IDO1*, *TIGIT*, *TIM-3*, and *LAG3*, which participate in T cell dysfunction and malfunction of anti-tumor immunity [51], and found that they were significantly elevated in ERcluster B and GeneCluster B. Despite higher infiltration of CD8+ T cells and gamma delta T cells in ERcluster B and GeneCluster B, which are usually regarded as a positive regulation of anti-tumor immunity and signs of a good prognosis, and considering the TME as a complicated and elaborate regulation network, we speculate that the overall effect of ER stress is closely correlated with T cell exhaustion and formation of an immunosuppressive TME in CRC. These results are in accordance with those of previous studies. For instance, Mohamed et al. [45] reported that the *PERK* pathway is involved in the immunosuppressive function of MDSC, and Cubillos-Ruiz et al. [19] illustrated that IRE1α-XBP1 axis activation in DCs leads to antigen-presenting dysfunction and corrupted antitumor effects of T cells in ovarian cancer. Our data also revealed that ERcluster B and GeneCluster B had a higher proportion of patients with dMMR. Currently, higher lymphocyte infiltration in the TME (“hot tumor”), increased expression of immune checkpoint genes, and MSI-H/dMMR are considered markers of a good response to immunotherapy [2,52,53]. Given the above, ER stress-related gene patterns (described as ERcluster B and GeneCluster B) might be a good indicator of response to immunotherapy. Moreover, an in vivo study demonstrated that *PERK* inhibitors increased tumor control and extended survival in combination with anti-PD-1 therapy [54], indicating that novel drugs targeting ER stress-related pathways might reverse the immunosuppressive TME and enhance the efficacy of immunotherapy.

Considering the clinical significance of ER stress-related gene patterns. We built a risk score using these ER stress-related genes to predict the survival of patients with CRC. After univariate, Lasso, and multivariate Cox regression analyses, 14 genes (*ASNS, CALR3, DNAJB2, EIF2AK4, ERMP1, FBXO6, FLOT1, HERPUD1, HYOU1, PDX1, SEC31A, TSPYL2, WIPI1,* and *YOD1*) that correlated with prognosis were identified and utilized in the construction of the risk score. Patients with a higher risk score were shown to have unfavorable outcomes, and the risk score was validated in TCGA COAD/READ patients. Next, we constructed a nomogram combining the risk score with clinical features to better predict patient survival with easier accessibility. As a result, the nomogram demonstrated superior ability in predicting the survival of patients with CRC. Moreover, we conducted a multi-omics analysis of these 14 critical ER stress-related risk score-building genes and found that most of them were differentially expressed in tumors. Among the 14 genes, *ASNS*, known as asparagine synthetase, has been shown to promote colon cancer progression through increasing asparagine level [55]; *EIF2AK4*, a risk factor identified in our study, has been shown to be a promising target in CRC treatment [56]; and *YOD1* was shown to participate in gallbladder cancer progression [57]. Our results confirmed these studies, and using the risk score of these 14 genes, those with elevated ER stress-related gene patterns might be identified and could be better treated with the combination of immunotherapy. Of note, future research might focus on the 14 genes regarding their roles in ER stress-related pathways, tumor progression, and remodeling of the CRC TME.

Furthermore, ST data of two tumor sections from two untreated CRC patients were utilized in the analysis of ER stress-related gene patterns at a quantitative spatial level. It has been demonstrated that CRC tumors have an activated ER stress phenotype. In addition, MDSC and Tregs have been validated to be associated with the activation of ER stress. Intriguingly, by applying the DEGs to the enrichment analysis, we found that the DEGs signature was significantly correlated with CAFs. Multiple studies have demonstrated that CAFs are associated with tumor progression, chemoresistance, and immunosuppression in the TME [58,59,60]. As described above, stromal cells, such as CAFs, were also found to be highly infiltrating in ERcluster B and GeneCluster B. Thus, we speculate that the ER stress-related gene pattern might participate in or regulate the infiltration and functions of CAFs, which further reinforces the formation of an immunosuppressive TME. These results shed some light on the relationship between ER stress and CAFs, but the underlying regulatory mechanisms are still largely unknown and future work might be focused on how ER stress-related pathways (e.g., *IRE1α*, *EIF2AK3*, and *ATF6* pathways) regulate CAFs in CRC TME and the role of CAFs in influencing CRC progression.

In conclusion, we identified two ER stress-related subtypes of CRC with distinct immune cell infiltration patterns and clinical features. ER stress was found to promote tumor progression and participate in the immunosuppression of the TME. Furthermore, we constructed a risk score and nomogram system for predicting CRC survival. We proposed a novel insight into the role of ER stress in CRC development and cell infiltration in the TME. To the best of our knowledge, this is the first comprehensive analysis of ER stress-related gene patterns in CRC tumor development and TME cell infiltration. However, several limitations exist. Firstly, in-depth cell subtype-specific analysis of ER stress-related gene patterns was confined due to the innate limitations of bulk-sequencing data and small samples of ST data. Secondly, the expression profile of CRC patients who received immunotherapy is lacking; thus, we could not test the subtypes or risk score in predicting immunotherapy response in patients with CRC. In the future, drugs that target ER stress may be combined with traditional therapy or immunotherapy for CRC treatment.

## Figures and Tables

**Table 1:** Parameters used in the construction of ER stress-related risk score.

Gene	Coef
*ASNS*	0.688343247
*CALR3*	−0.572927078
*DNAJB2*	0.455677169
*EIF2AK4*	0.831306602
*ERMP1*	0.764514094
*FBXO6*	−0.388996026
*FLOT1*	0.463952998
*HERPUD1*	0.803594073
*HYOU1*	0.233149469
*PDX1*	−0.60035933
*SEC31A*	0.493525152
*TSPYL2*	0.646208663
*WIPI1*	−0.244404952
*YOD1*	0.292412602

**Table 2:** Characteristics of CRC patients in ERcluster A and ERcluster B in GEO datasets.

Characteristics	ERcluster A (*n* = 538)	ERcluster B (*n* = 346)	Total (*n* = 884)	*p* Value
**Age**				0.84
<65	183 (34.01%)	111 (32.08%)	294 (33.26%)	
≥65	281 (52.23%)	186 (53.76%)	467 (52.83%)	
Unclear	74 (13.76%)	49 (14.16%)	123 (13.91%)	
**Gender**				0.63
Female	203 (37.73%)	141 (40.75%)	344 (38.91%)	
Male	261 (48.51%)	157 (45.38%)	418 (47.29%)	
Unclear	74 (13.76%)	48 (13.87%)	122 (13.80%)	
**Stage**				0.17
I	61 (11.34%)	23 (6.65%)	84 (9.50%)	
II	221 (41.08%)	142 (41.04%)	363 (41.06%)	
III	176 (32.71%)	130 (37.57%)	306 (34.62%)	
IV	79 (14.68%)	50 (14.45%)	129 (14.59%)	
Unclear	1 (0.19%)	1 (0.29%)	2 (0.23%)	
**Grade**				0.18
1	10 (1.86%)	6 (1.73%)	16 (1.81%)	
2	84 (15.61%)	50 (14.45%)	134 (15.16%)	
3	11 (2.04%)	16 (4.62%)	27 (3.05%)	
Unclear	433 (80.49%)	274 (79.20%)	707 (79.98%)	
**Tumor_location**				6.20 × 10^−10^
Distal	253 (47.03%)	98 (28.32%)	351 (39.71%)	
Proximal	105 (19.52%)	127 (36.71%)	232 (26.24%)	
Unclear	180 (33.45%)	121 (34.97%)	301 (34.05%)	
***KRAS*_mutation**				0.13
No	188 (34.94%)	140 (40.46%)	328 (37.10%)	
Yes	143 (26.58%)	74 (21.39%)	217 (24.55%)	
Unclear	207 (38.48%)	132 (38.15%)	339 (38.35%)	
***BRAF*_mutation**				3.20 × 10^−14^
No	307 (57.06%)	154 (44.51%)	461 (52.15%)	
Yes	5 (0.93%)	46 (13.29%)	51 (5.77%)	
Unclear	226 (42.01%)	146 (42.20%)	372 (42.08%)	
**MSI_status**				2.20 × 10^−17^
dMMR	14 (2.60%)	63 (18.20%)	77 (8.71%)	
pMMR	322 (59.85%)	137 (39.60%)	459 (51.92%)	
Unclear	202 (37.55%)	146 (42.20%)	348 (39.37%)	
**Adjuvant_chemotherapy**				0.38
No	194 (36.06%)	132 (38.15%)	326 (36.88%)	
Yes	155 (28.81%)	85 (24.57%)	240 (27.15%)	
Unclear	189 (35.13%)	129 (37.28%)	318 (35.97%)	

dMMR: deficient mismatch repair; pMMR: proficient mismatch repair.

## Data Availability

Data related to gene expression, and clinical information of TCGA COAD/READ are available from GDC data portal (https://portal.gdc.cancer.gov). GSE17536, GSE38832, and GSE39582 datasets can be downloaded from the Gene Expression Omnibus (GEO) (https://www.ncbi.nlm.nih.gov/geo/), data included in this study were accessed on 1 November 2021.

## Supplementary Materials

The following supporting information can be downloaded at: https://www.mdpi.com/article/10.3390/cancers14143326/s1, Supplementary Figure S1. Unsupervised clustering of ER stress-related genes; Supplementary Figure S2. Abundance estimation of infiltration cells in the TME using an MCP-counter algorithm; Supplementary Figure S3. Unsupervised clustering of DEGs between ERcluster A and B; Supplementary Figure S4. Identification of prognostic genes and validation of the risk score in testing group; Supplementary Figure S5. Correlation evaluation of infiltration cells with risk score; Supplementary Figure S6. Common drug sensitivity between high- and low-risk groups; Supplementary Figure S7. RT-qPCR results of the 14 risk score-building genes between normal adjacent and tumor tissues; Supplementary Figure S8. Identification of protein-protein interaction modules within the 232 prognostic genes; Supplementary Table S1. Characteristics of CRC patients in GeneCluster A and GeneCluster B in GEO datasets; Supplementary Table S2. 232 genes with prognostic significances in the Univariate Cox analysis; Supplementary Table S3. 268 ER stress-related genes; Supplementary Table S4. DEGs between ERcluster A and B; Supplementary Table S5. Forward and reverse primer sequences; Supplementary Table S6. Clinical characteristics of 884 CRC patients from GSE17536, GSE38832, and GSE39582 datasets; Supplementary Table S7. ER stress-related ERcluster and GeneCluster in GEO datasets; Supplementary Table S8. Functional analysis of DEGs between ERcluster A and ERcluster B.
