# Supplementary material for: Identification of Endoplasmic Reticulum Stress-Related Subtypes, Infiltration Analysis of Tumor Microenvironment, and Construction of a Prognostic Model in Colorectal Cancer
Source: Cancers (Basel). 2022 Jul 8;14(14):3326. doi: 10.3390/cancers14143326 (PMC9322646; doi:10.3390/cancers14143326)
Supplement: Supplementary file 1 [file cancers-14-03326-s001.zip › cancers-1771022-supplementary figures.pdf]

Identification of Endoplasmic Reticulum Stress-Related Subtypes, Infiltration Analysis of Tumor Microenvironment, and Construction of a Prognostic Model in Colorectal Cancer

Baike Liu et al.
Supplementary Figures

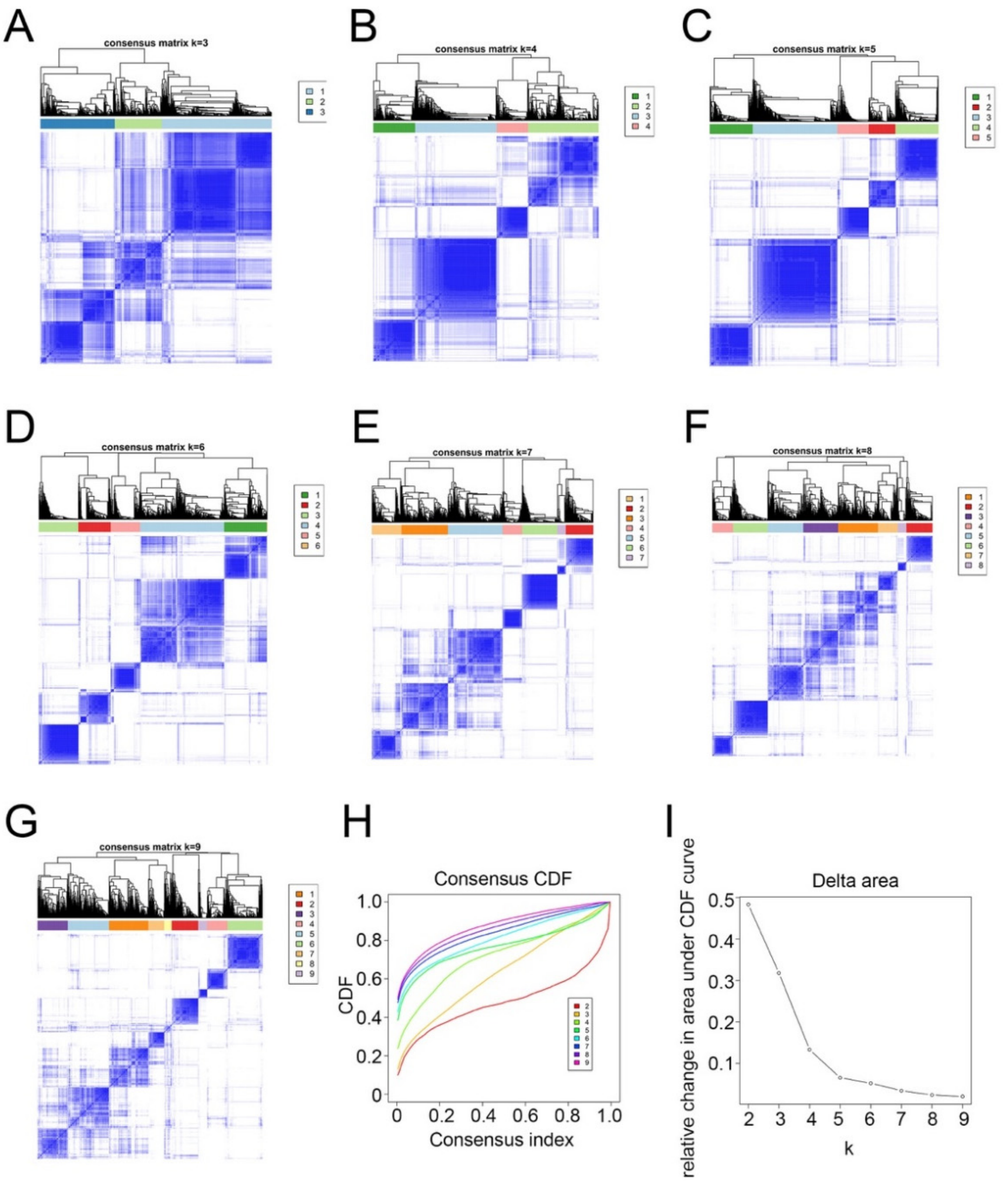

Supplementary Figure S1. Unsupervised clustering of ER stress-related genes. (**A**–**G**) Consensus matrix heatmaps for k = 3–9. (**H**,**I**) Empirical cumulative distribution function (CDF) and relative change area under CDF curve of each k value.

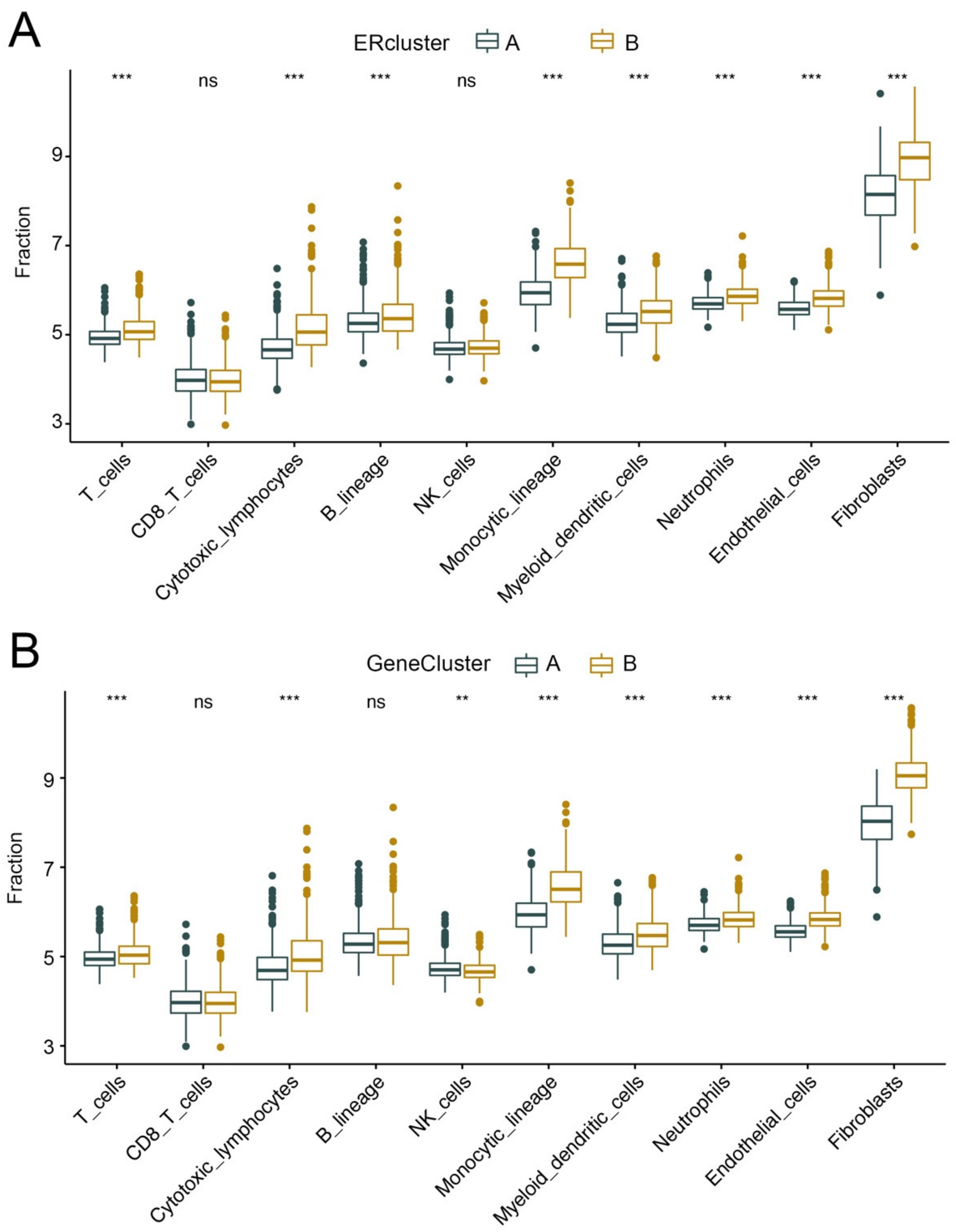

Supplementary Figure S2. Abundance estimation of infiltration cells in the TME using an MCP-counter algorithm. (**A**) Cell infiltration patterns between ERcluster A and B (*ns* means not significant, *** $p < 0.001$). (**B**) Cell infiltration patterns between GeneCluster A and B (*ns* means not significant, ** $p < 0.01$, *** $p < 0.001$).

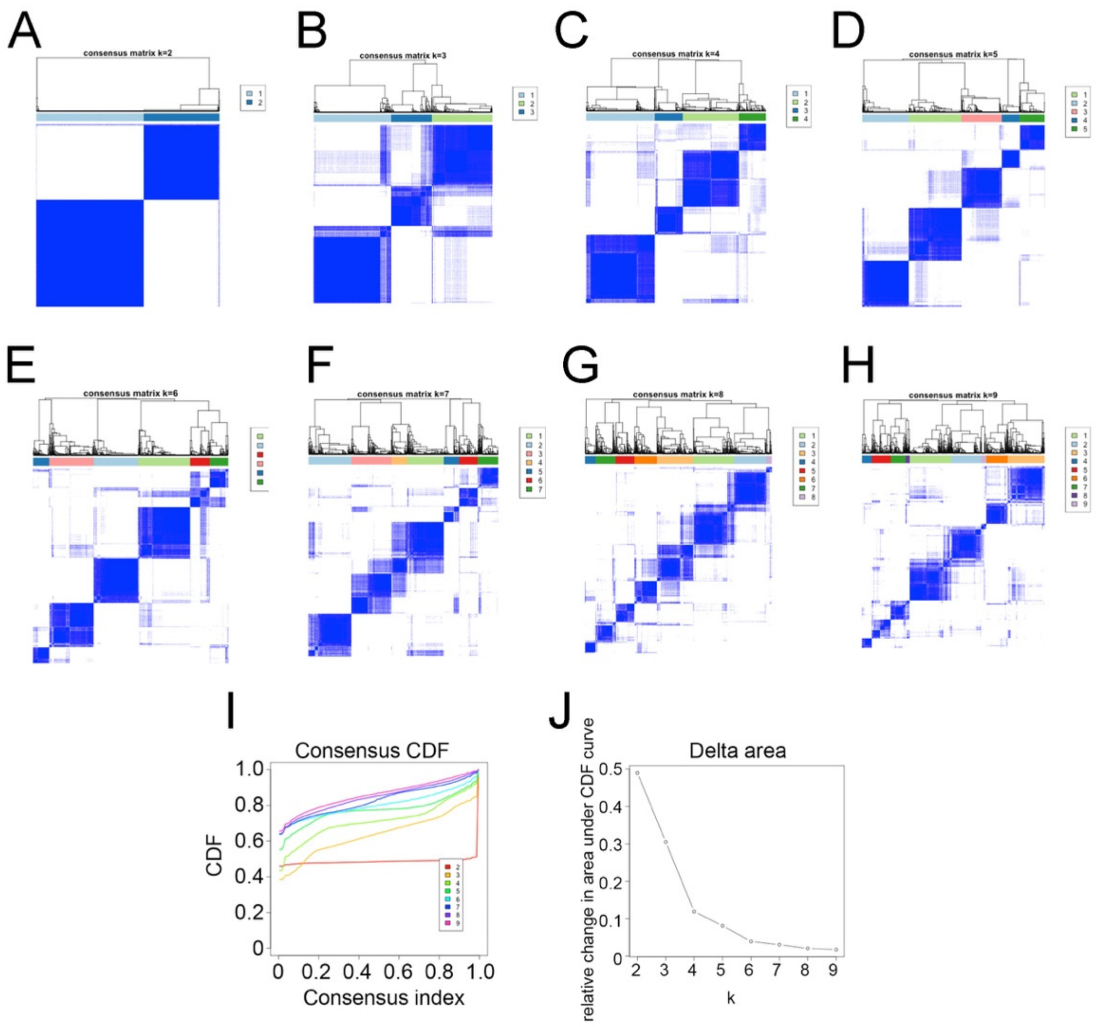

Supplementary Figure S3. Unsupervised clustering of DEGs between ERcluster A and B. (**A–H**) Consensus matrix heatmaps for k = 2–9. (**I**,**J**). Empirical CDF and relative change area under CDF curve of each k value.

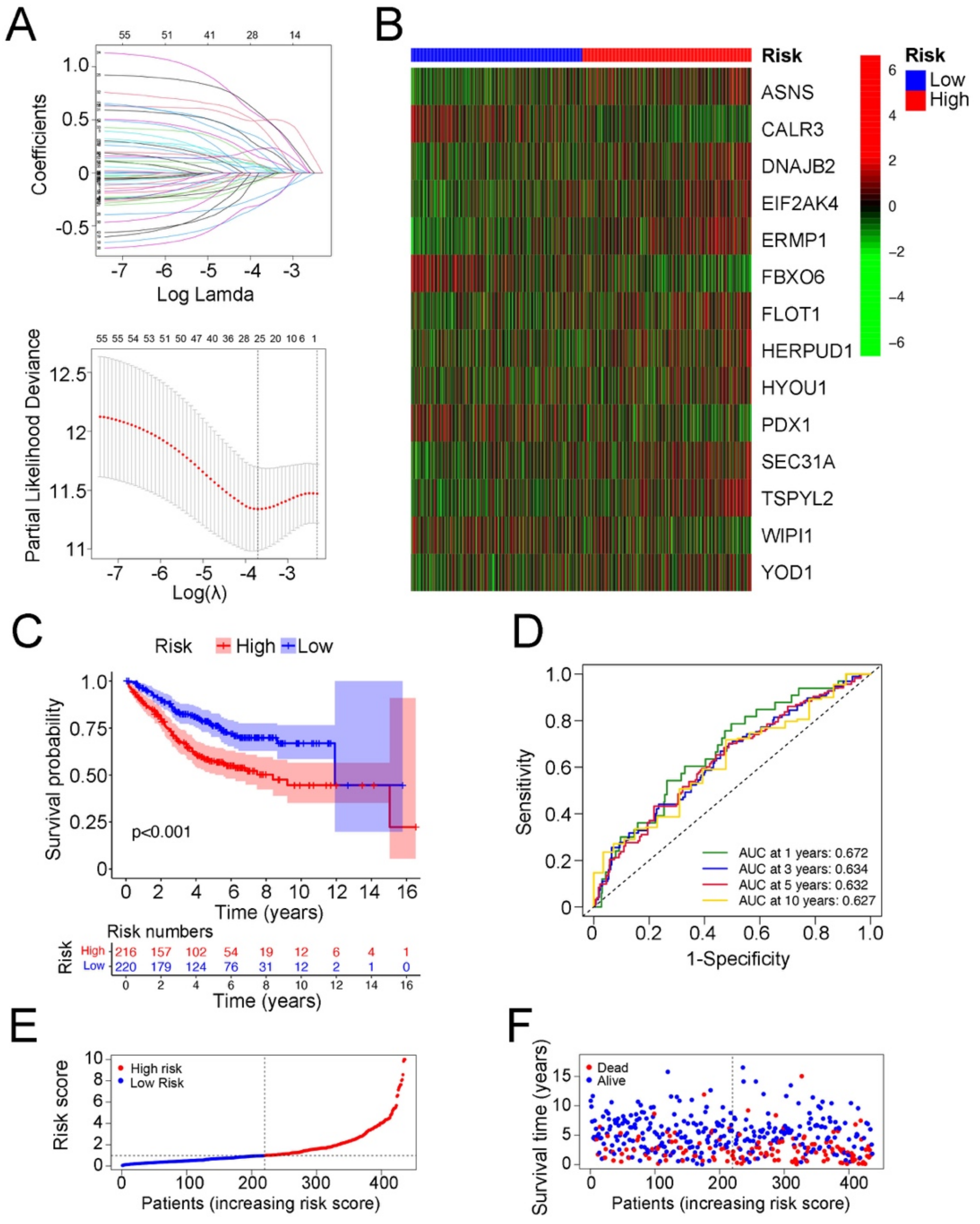

Supplementary Figure S4. Identification of prognostic genes and validation of the risk score in testing group. (**A**,**B**) The coefficient profiles and partial likelihood deviance of the LASSO regression model. (**C**) heatmap of the 14 risk score-building genes. (**D**) Survival prediction of the risk score in the testing group. (**E**) ROC curves of the survival prediction using risk score in the testing group. (**F**,**G**) Ranked plots and distribution plots of risk score and clinical outcomes in the testing group.

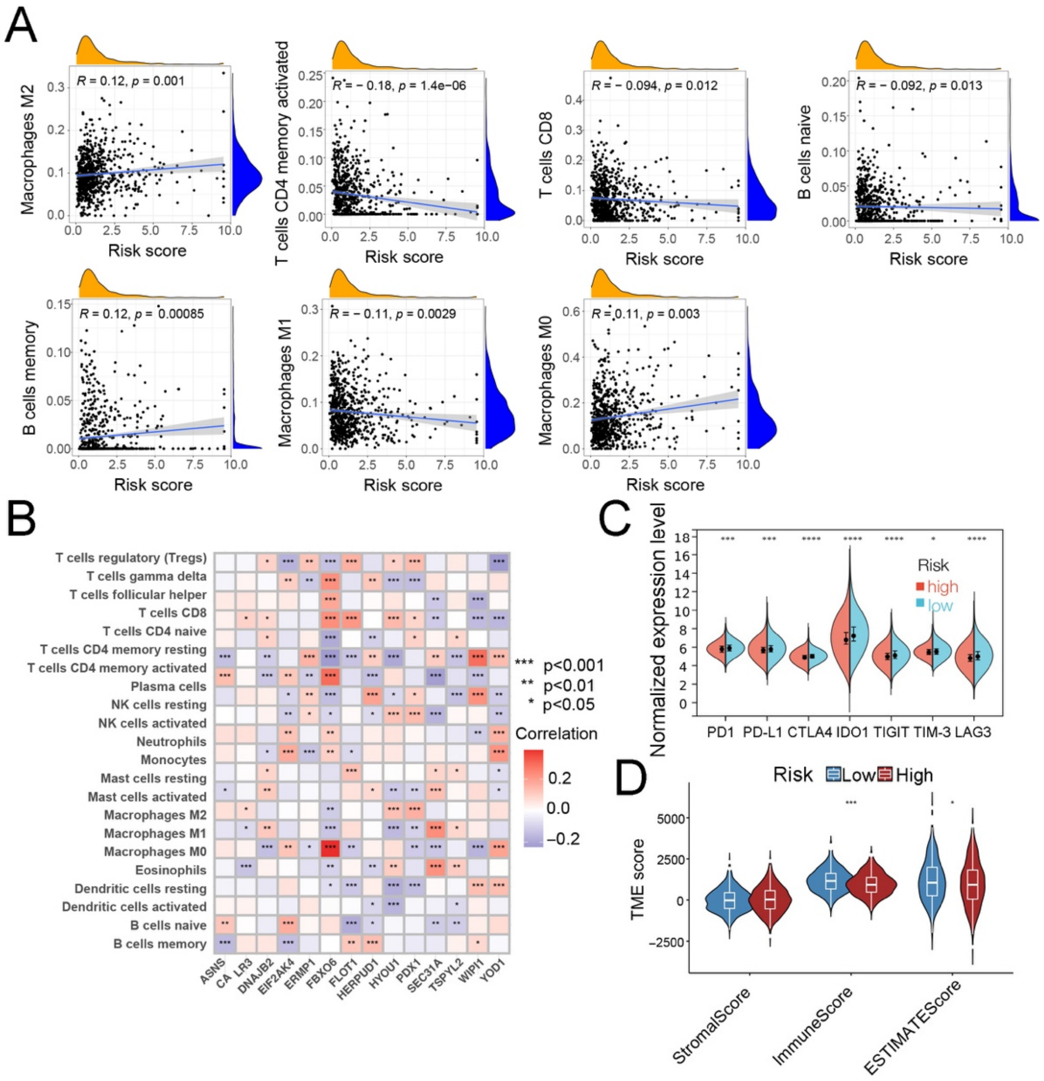

Supplementary Figure S5. Correlation evaluation of infiltration cells with risk score. (**A**) Correlation between risk score and immune cell types. (**B**) Correlation between the abundance of immune cells and 14 risk score-building genes in the CRC samples. (**C**) Expression level of common immune checkpoint genes (PD1, PD-L1, CTLA4, IDO1, TIGIT, TIM-3, LAG3) between high- and low-risk group (* $p < 0.05$, *** $p < 0.001$, **** $p < 0.0001$). (**D**) ESTIMATE results of immune and stromal scores between high- and low-risk group (* $p < 0.05$, *** $p < 0.001$).

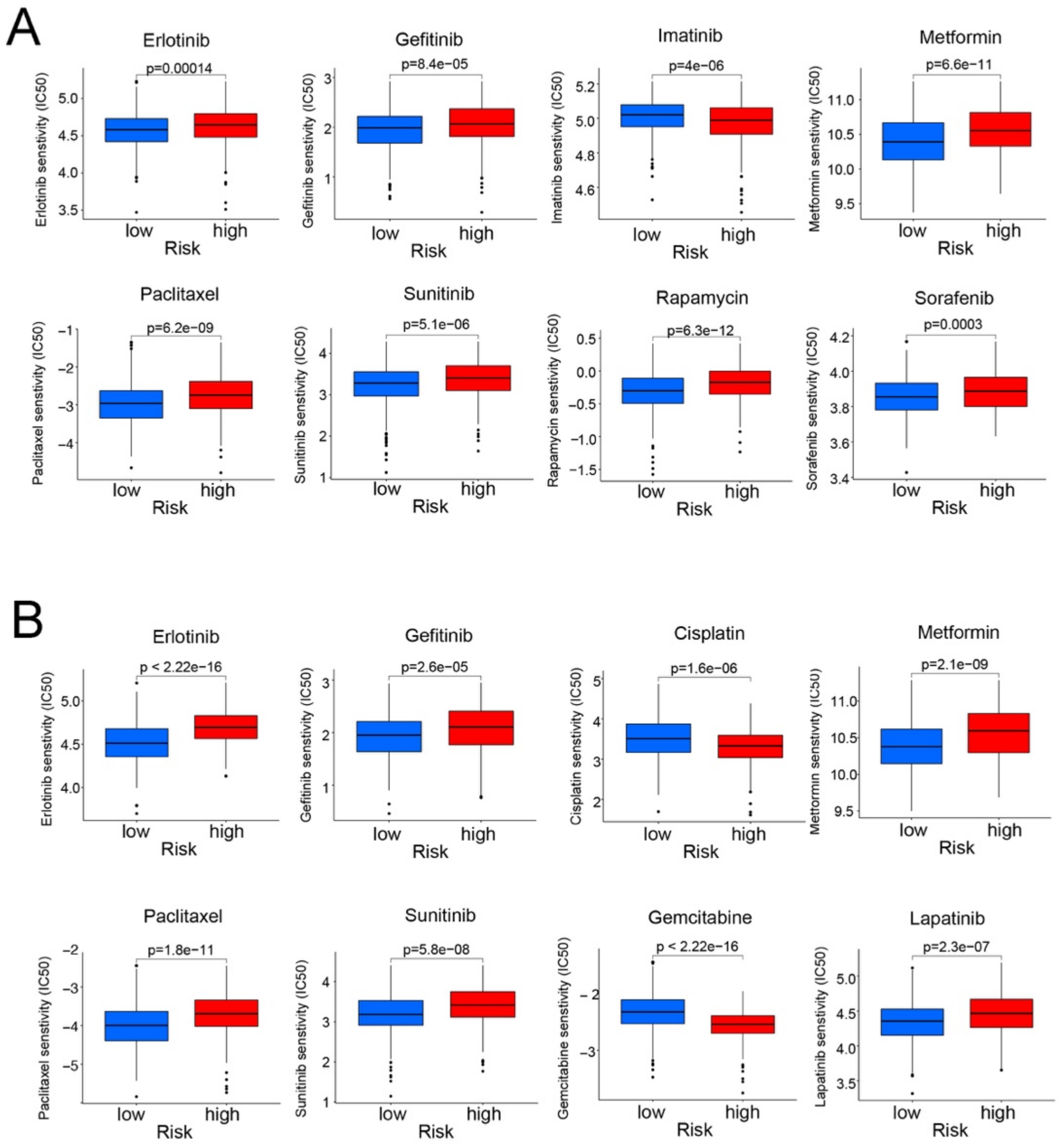

Supplementary Figure S6. Common drug sensitivity between high- and low-risk groups. (**A**) Drug sensitivity analysis using GEO CRC datasets. (**B**) Drug sensitivity analysis using TCGA COAD/READ cohorts.

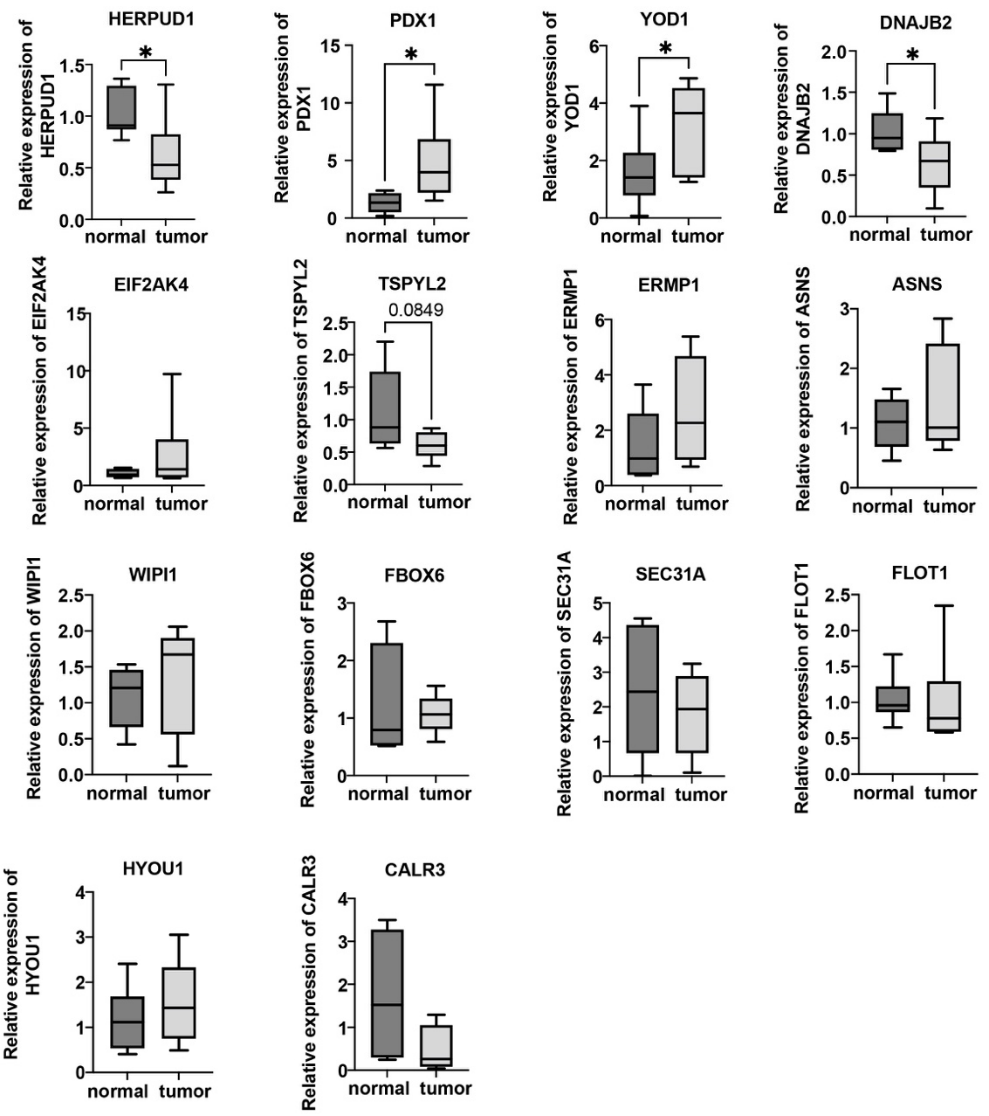

Supplementary Figure S7. RT-qPCR results of the 14 risk score-building genes between normal adjacent and tumor tissues (* $p < 0.05$).

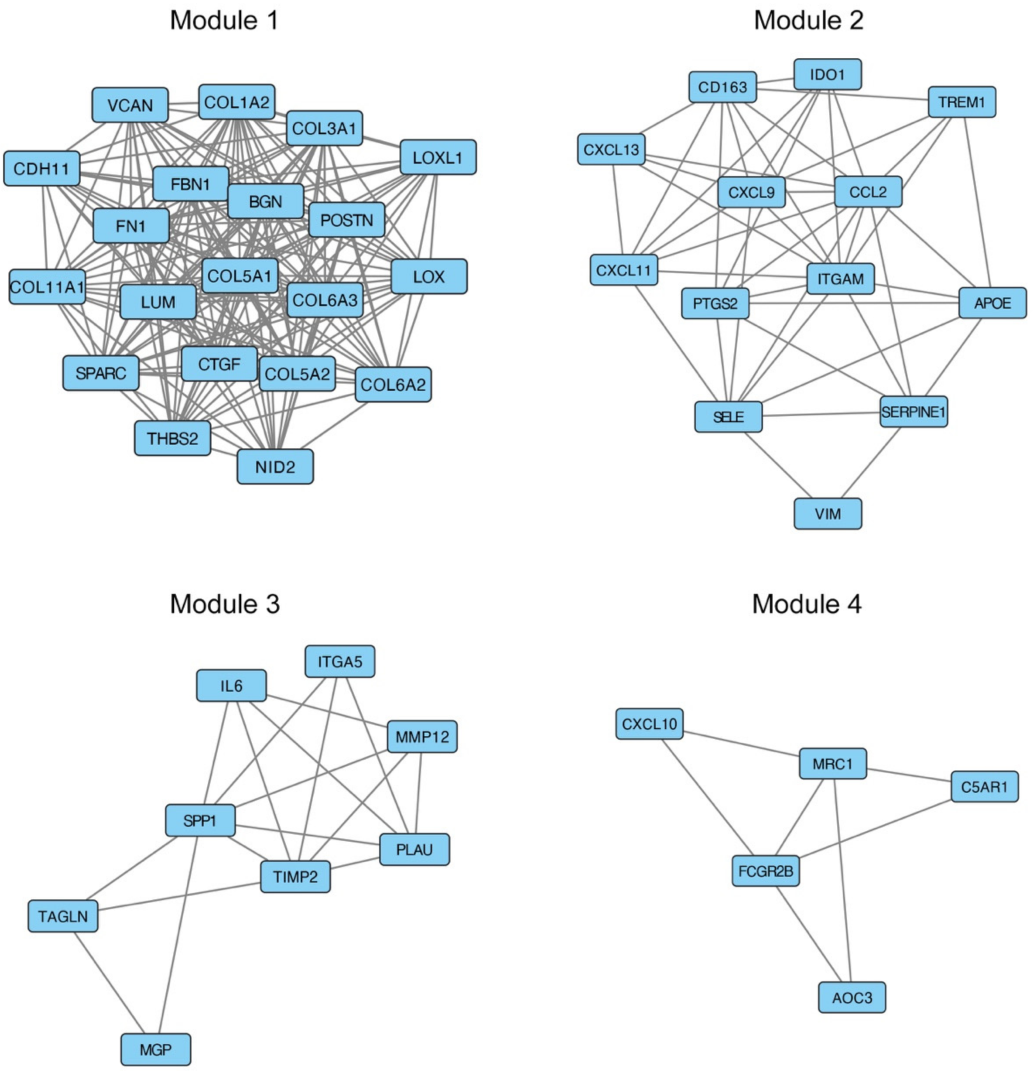

Supplementary Figure S8. Identification of protein-protein interaction modules within the 232 prognostic genes (GOBP_RESPONSE_TO_ENDOPLASMIC_RETICULUM_STRESS).
